# Evaluation of Antibacterial and Antifungal Effects of Calcium Hydroxide Mixed with Two Different Essential Oils

**DOI:** 10.3390/molecules27092635

**Published:** 2022-04-20

**Authors:** Gokalp Cosan, Cenk Serhan Ozverel, Duygu Yigit Hanoglu, Kemal Husnu Can Baser, Yasar Meric Tunca

**Affiliations:** 1 Department of Endodontics, Faculty of Dentistry, Near East University, Nicosia 99138, Cyprus; gokalp.cosan@neu.edu.tr; 2 Department of Basic Medical Sciences, Faculty of Dentistry, Near East University, Nicosia 99138, Cyprus; cenkserhan.ozverel@neu.edu.tr; 3 Desam Institute, Near East University, Nicosia 99138, Cyprus; 4 Department of Pharmaceutical Botany, Faculty of Pharmacy, Near East University, Nicosia 99138, Cyprus; duygu.yigithanoglu@neu.edu.tr; 5 Department of Pharmacognosy, Faculty of Pharmacy, Near East University, Nicosia 99138, Cyprus; kemalhusnucan.baser@neu.edu.tr; 6 Department of Endodontics, Faculty of Dentistry, University of Kyrenia, Nicosia 99138, Cyprus

**Keywords:** endodontics, essential oils, antimicrobial, antibacterial, *E. faecalis*, *C. albicans*, calcium hydroxide

## Abstract

Background: Calcium hydroxide is a routinely used material for root canal disinfection during root canal treatment. Natural products have great potential in terms of their antibacterial effects. This study aimed to establish an effective alternative intracanal medicament using *Origanum dubium (O. dubium)* and *Mentha spicata (M. spicata)* essential oils. Materials and Methods: *O. dubium* and *M. spicata,* collected from Lefke, Cyprus, were separately subjected to hydrodistillation. The obtained essential oil compositions were analysed simultaneously by gas chromatography (GC) and gas chromatography/mass spectrometry (GC-MS). The compositions were then divided into groups and mixed with calcium hydroxide at a 1:1 concentration; after that, the pastes were tested on *Enterococcus faecalis* (*E. faecalis*) and *Candida albicans (C. albicans),* which are the most common resistant pathogenic microorganisms in the root canal. The antibacterial activity of the pastes was measured using a disk diffusion assay. Results: The GC and GC-MS analyses revealed that *O. dubium* and *M. spicata* had major compositions of carvacrol (75.8%) and carvone (71.3%), respectively. Antimicrobial activity was found to be significantly higher when study groups with *O. dubium* essential oil were applied to both *E. faecalis* and *C. albicans*. The results also show that *M. spicata,* together with calcium hydroxide, demonstrated a significant antifungal effect on *C. albicans* when incubated for 72 h. Conclusions: *M. spicata* was found to be an effective antimicrobial agent on *C. albicans,* whereas *O. dubium* was found to be very effective on both *E. faecalis* and *C. albicans*. These data demonstrate that these natural essential oils may be promising candidates for alternative intracanal medicament in future routine clinical applications.

## 1. Introduction

The removal of microorganisms from the root canal system is a crucial step in any successful endodontic treatment [1,2]. In particular, if living microorganisms are not eliminated effectively, their presence may lead to a resistant infection and poor healing [1]. In dentistry, *E. faecalis* is a particularly common Gram-positive bacteria in root canals diagnosed with apical periodontitis, and it has been observed to be one of the main pathogens in secondary endodontic infections [2]. *E. faecalis* can survive in very harsh nutrient-deficient environments with a high alkaline pH reaching 11.5. *E. faecalis* has the capacity to grow as a biofilm on the root canal walls and can cause a mono-infection in treated canals without any synergistic support from other pathogen species. In addition, *E. faecalis* is known for its high resistance to antimicrobial agents applied during root canal therapy [3,4]. However, *E. faecalis* is not the only pathogen known to be associated with unsuccessful root canal treatment. *C. albicans* is one of the most common commensals and pathological fungi present in the oral cavity [5]. *C. albicans* is a round/oval-shaped gram-positive yeast-like fungus that is commonly found in 7–18% of unsuccessful root canal treatments due to tenacious or secondary endodontic infections related to recurrent periradicular lesions [6].

Several procedures are applied during endodontic treatment to overcome the aforementioned problems. Instrumentation and irrigation are just some of the procedures that have been shown to have a great effect on the success of root canal treatment. It is widely known that intracanal medication provides the best canal disinfection [7]. The ideal use of intracanal medicaments can provide disinfection by significantly reducing the amount of microorganisms and their by-products in the root canal system. Disinfecting root canals is also essential for preventing tissue damage caused by microorganisms and their by-products [8,9]. For this purpose, the most commonly used disinfection material in the clinic today is calcium hydroxide [10].

Calcium hydroxide is an odourless white powder with the chemical formula Ca(OH)_2_ and a molecular weight of 74.09. Chemically, it is known to be a strong base that is in contact with aqueous liquids (pH about 12.5–12.8) [11]. Calcium hydroxide exerts antimicrobial activity by dissociating into calcium and hydroxyl ions. These ions are highly oxidant anions and show extreme reactivity with several biomolecules [12]. The main effects of calcium hydroxide are attributed to the action of these ions, which raise the pH [13]. The alkaline environment detoxifies bacterial lipopolysaccharides (LPS) by removing esterified fatty acids and altering their chemical conformation. As a result, bacterial cytoplasmic membrane integrity is destroyed [11,12,13,14,15]. These hydroxyl ions exert an antimicrobial effect after diffusion through the entire root canal system via direct or indirect contact [16]. However, calcium hydroxide is not equally effective against all types of bacteria found in root canals [17]. For instance, the fact that *E. faecalis* is resistant to calcium hydroxide has led researchers to investigate new antimicrobial agents and their effects as alternatives [18]. Furthermore, *C. albicans*, one of the most common yeasts found in the oral cavity, has also exhibited some resistance. According to the results of a recent study conducted in 2022, calcium hydroxide has been reported to be ineffective against this most resistant fungal species of *C. albicans*. Therefore, new alternative treatment methods are required for routine endodontic treatment protocols [19,20].

Throughout history, it has been possible to treat many diseases with plants and their products collected from nature. Natural products, especially essential oils, are known for their antimicrobial activity [21]. Essential oils are very effective in opposition to both Gram-positive and Gram-negative bacteria, as well as many other fungal species, including *C. albicans* [22]. Some plants known to produce such oils are Cyprus mountain thyme (*Origanum dubium* Boiss., or *O. dubium*) and Cyprus garden mint (*Mentha spicata* L., or *M. spicata*). The essential oils extracted from these plants are known to have great antimicrobial potential. Carvacrol, which is found in oregano and thyme, has also been established as a great inducer of antimicrobial activity, making essential oils bearing it great candidates for future use in root disinfection procedures. Carvacrol exerts antimicrobial effects by blocking ATPase activity and increasing the permeability of bacterial cell membranes. Carvacrol not only prevents microbial colonisation but also increases the susceptibility of pathogens to antibacterial agents [23].

This research aimed to analyse the in vitro antimicrobial effects of two different essential oils (*O. dubium* and *M. spicata*) either combined with calcium hydroxide or used alone against the two most common resistant microorganisms found in the oral cavity (*E. faecalis* and *C. albicans*). This in vitro study provides preliminary data on potential intracanal drug combinations that can be used to prevent the failure of endodontic treatment. The null hypotheses of the study were as follows: (1) *O. dubium* essential oil would not have an antimicrobial effect on either *E. faecalis* or *C. albicans* and (2) *M. spicata* essential oil would not have an antimicrobial effect on either *E. faecalis* or *C. albicans.*

## 2. Results

Gas chromatography and mass spectrometry data analysis revealed 33 compounds characterised by the essential oil composition of *M. spicata* (Table 1). The major compounds were carvone (71.3%), followed by limonene (12.5%) and 1,8-cineole (3.6%). Another 27 compounds were recognised in the *O. dubium* essential oil, of which the major compound was carvacrol (75.8%; Table 2).

**Figure 1:**
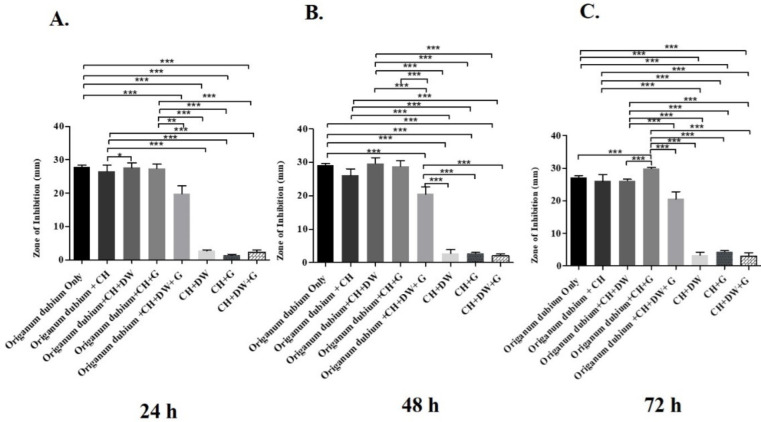
Inhibition zone diameters (mm) of different study groups containing the essential oil of *O. dubium* on *E. faecalis*. (**A**). Inhibition zone diameters (mm) measured 24 h post-incubation with various groups on *E. faecalis*. (**B**) Inhibition zone diameters (mm) measured 48 h post-incubation with various groups on *E. faecalis*. (**C**) Inhibition zone diameters (mm) measured 72 h post-incubation with various groups on *E. faecalis* (CH: calcium hydroxide; G: glycerin, DW: distilled water). Values represent mean zone of inhibition ± standard deviation (* *p* < 0.05, ** *p* < 0.01, *** *p* < 0.001).

**Figure 2:**
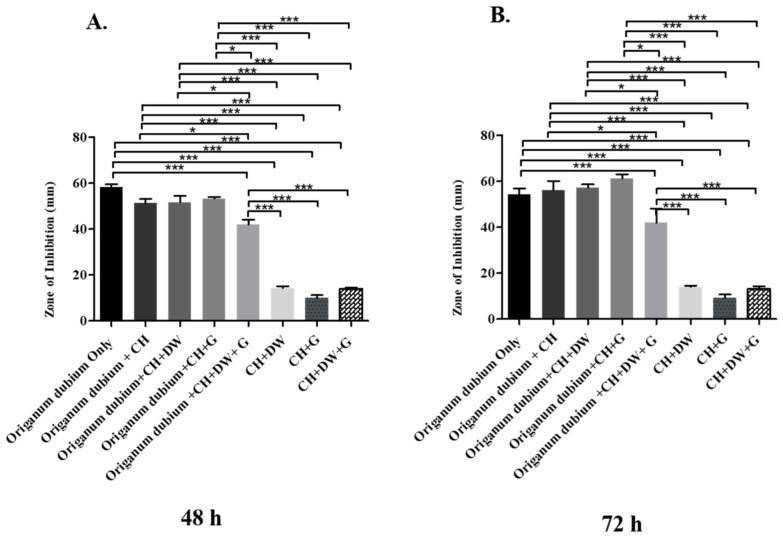
Inhibition zone diameters (mm) of different study groups containing the essential oil of *O. dubium* on *C. albicans*. (**A**) Inhibition zone diameters (mm) measured 48 h post-incubation with various groups on *C. albicans*. (**B**) Inhibition zone diameters (mm) measured 72 h post-incubation with various groups on *C. albicans* (CH: calcium hydroxide; G: glycerin, DW: distilled water). Values represent mean zone of inhibition ± standard deviation (* *p* < 0.05, *** *p* < 0.001).

**Figure 3:**
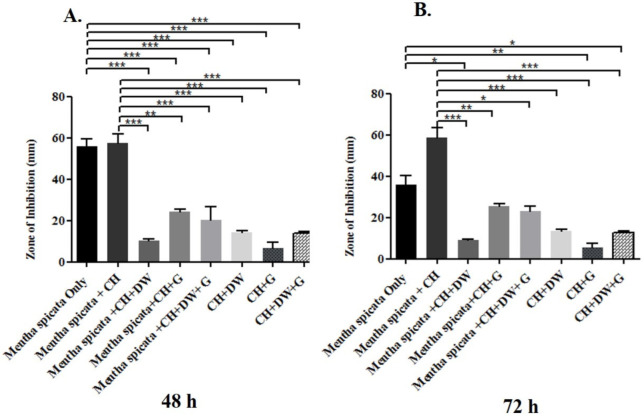
Inhibition zone diameters (mm) of different study groups containing the essential oil of *M. spicata* on *C. albicans*. (**A**) Inhibition zone diameters (mm) measured 48 h post-incubation with various groups on *C. albicans*. (**B**) Inhibition zone diameters (mm) measured 72 h post-incubation with various groups on *C. albicans*. (CH: calcium hydroxide; G: glycerin, DW: distilled water). Values represent mean zone of inhibition ± standard deviation (* *p* < 0.05, ** *p* < 0.01, *** *p* < 0.001).

The data in Figure 1, Figure 2 and Figure 3 describe the inhibition zone measurements (mm) of the different study groups and indicate relative statistical differences. The addition of *O. dubium* essential oil to the calcium hydroxide groups containing either distilled water or glycerin showed antimicrobial activity on both *C. albicans* and *E. faecalis*, whereas the *M. spicata* extract exerted antimicrobial activity only on *C. albicans*.

The antimicrobial activity study on *E. faecalis* was performed at three different time intervals (24 h, 48 h, and 72 h; Figure 1). At all three intervals, the addition of *O. dubium* essential oil significantly increased the antimicrobial activity regardless of the addition of either distilled water, glycerin, or both to the calcium hydroxide. Similar trends in activity were observed in all the different intragroup comparisons at all time intervals (Figure 1). A combination of calcium hydroxide, glycerin, and *O. dubium* oil demonstrated the significantly highest level of antimicrobial activity, with the formation of more than a 30 mm inhibition zone on *E. faecalis* (*p* < 0.001) when compared with all other combination groups at 72 h (Figure 1C). For the 24 h and 48 h measurements, intergroup comparisons of the trios of (1) calcium hydroxide, glycerin, and *O. dubium* essential oil and (2) calcium hydroxide, distilled water, and *O. dubium* essential oil did not show any significant differences in antimicrobial activity against *E. faecalis* (Figure 1A,B). Furthermore, the level of antimicrobial activity did not exhibit any significant difference in the calcium hydroxide groups in the absence of any essential oil (Figure 1). 

The antimicrobial effect of the essential oil of *O. dubium* added to the different calcium hydroxide study groups was further investigated with *C. albicans*. The effect on *C. albicans* was studied at two different time intervals, as there was no effective growth of microorganisms within 24 h (48 h and 72 h; Figure 2). The antimicrobial activity of the different *O. dubium* groups on *C. albicans* was found to be higher when compared with the corresponding activity on *E. faecalis* in all the different study groups (Figure 1 and Figure 2). General trends of antimicrobial activity were found to be significantly higher when the *O. dubium* essential oil was added to the calcium hydroxide groups in comparison with the groups not containing *O. dubium* at both time intervals (48 h and 72 h; *p* < 0.001). At both time intervals, relatively similar trends of antimicrobial activity were observed in all study groups except for the one containing calcium hydroxide, glycerin, and *O. dubium*. At 48 h, the zone of inhibition formed by the calcium hydroxide, glycerin, and *O. dubium* oil group was reported to be significantly higher than that of any other groups that did not have essential oil at 48 h (Figure 2B). Furthermore, at the same time interval, the calcium hydroxide, glycerin, and *O. dubium* essential oil groups had the highest antimicrobial activity when compared to all other study groups (Figure 2B). 

*M. spicata* is another essential oil that was investigated in this study. The study indicated that the essential oil of *M. spicata* did not have any antimicrobial effect on *E. faecalis* and that it did not show any inhibition zone (mm) in the bacteria culture. However, *M. spicata* demonstrated a remarkable zone of inhibition in the *C. albicans* culture. The combination of *M. spicata* oil and calcium hydroxide demonstrated the significantly highest zone of inhibition in comparison with other combination study groups at 72 h of incubation (*p* < 0.001; Figure 3B). The groups with (1) *M. spicata* essential oil, calcium hydroxide, and glycerin and (2) *M. spicata* essential oil, calcium hydroxide, glycerin, and distilled water induced slightly higher antimicrobial activity when compared with study groups that did not contain *M. spicata* essential oil at both time intervals (Figure 3A,B).

## 3. Discussion

The null hypothesis of the study for *O. dubium* was rejected. The essential oil of *O. dubium* exerted significant antimicrobial activity on both *E. faecalis* and *C. albicans*. The null hypothesis for *M. spicata* was partially rejected, as the oil showed a good antimicrobial effect on *C. albicans;* however, it did not exert any antimicrobial effect on *E. faecalis*.

The elimination of microorganisms from the root canal system, especially from dentin tubules, is key to successful endodontic therapy. These areas are considered a great niche for microorganisms, as they are low in oxygen concentration, thus promoting their growth. *C. albicans* is the most abundant fungus found in the oral cavity and often causes endodontic treatment failure [24]. The polymorphic nature of *C. albicans* gives the microorganism great superiority over neglected mucosal barriers, leading to the development of oral and disseminated infections. *E. faecalis* is one of the most resistant bacteria commonly found in root canal treatments [25]. Similar to *C. albicans*, *E. faecalis* is a major opportunistic bacterium that is of critical concern in immunocompromised patients [26]. Pinheiro et al. emphasised that *E. faecalis* was the most commonly isolated bacteria (45.8%) from root canal systems in formerly treated cases [27].

Although calcium hydroxide is used as an intracanal medication in endodontic treatments, there is still significant concern about its antimicrobial activity. Recent studies have demonstrated that calcium hydroxide alone may be less effective or inadequate when compared with other newly developed intracanal medications [28]. According to the results of a study conducted by Zancan et al., calcium hydroxide alone was found to be insufficient for eliminating bacteria [29]. In fact, it is widely known that the alkaline environment created by the high pH of calcium hydroxide is insufficient in destroying bacteria on its own. Bacteria can easily adapt to the alkaline environment formed as a result of their cytoplasmic activity. Specifically, Krishnamoorthy et al. observed that the properties of several *C. albicans* genes induce biofilm formation, tissue penetration, and tissue invasion. These activities were reported to significantly increase in the presence of *E. faecalis* [26]. In another study, Cook et al. investigated the quality of medications with or without calcium hydroxide application before root canal filling. They also compared the effectiveness of 2% chlorhexidine on dentinal tubules’ bacterial infections. The data indicated that the use of 2% chlorhexidine, followed by root canal filling, was more effective in disinfecting the bacterial colonies of *E. faecalis* compared with calcium hydroxide or immediate canal filling [30]. In a similar way, Peters et al. claimed that the number of Gram-positive bacteria decreased in the root canal system after dressing with calcium hydroxide [31]. Data reported in the literature have also demonstrated that calcium hydroxide is unsuccessful in removing bacteria from the root canal system [32]. Studies have also reported that seven days of application of calcium hydroxide is the minimum time of incubation for the successful elimination of bacteria, rather than administering the medication for 10 min. The presence of fungi constituted by *C. albicans* has been identified in primary root canal infections but is more frequent in unsuccessful endodontic treatments in comparison with other microorganisms. Their occurrence in secondary endodontic treatment failures varies between 1% and 17% [33,34]. This could be a result of the fact that *C. albicans* can live in a wide range of pH values, which explains its resistance to calcium hydroxide at high pH values. Moreover, calcium hydroxide further increases the concentration of Ca^2+^ ions that enhance *C. albicans* growth, demonstrating the limited or complete absence of its antifungal effect [35]. Together, these studies indicate that there is a need for an alternative disinfectant material for clinical use in endodontic treatment.

Natural products have been widely applied in traditional medicine, but their use is also highly popular in the fields of modern dentistry and medicine. Herbs with medicinal properties are useful and effective sources for the treatment of various disease processes. Although chemo-mechanical preparation of the root canal decreases the number of pathogens present, intracanal medicament with antibacterial action is required to maximise the disinfection of the root canal system [36]. Interest in essential oils, which are considered natural products, and their activity is increasing worldwide [14,37,38].

This research was conducted to investigate the potential of using *O. dubium* or *M. spicata* essential oils as intracanal dressings during root canal treatment, since the materials currently used have been reported to be insufficient in disinfection of root canals. The chemical components of the essential oils of *O. dubium* and *M. spicata* were used to determine their respective ingredients and to obtain a better understanding of their antibacterial and antifungal effects. In the present study, the major component of *O. dubium* essential oil was found to be carvacrol, with a ratio of 75.8%, and the major component of *M. spicata* essential oil was carvone, with a ratio of 71.3%; both of these values were determined by GC and GC-MS analysis.

Carvacrol is known as the main component in *Origanum* oils, but the ratio varies species-wise and according to the geographical region from which it is collected [39]. Carvacrol has a broad spectrum of antibacterial potential that is exerted by impeding ATPase activity and improving the nonselective permeability of bacterial cell membranes. The antibacterial effect of carvacrol against *E. faecalis* was demonstrated in a previous study carried out by Nosrat et al. [25]. This antimicrobial activity can be attributed to the disruption of bacterial cell membranes. Carvacrol not only disrupts bacterial cell walls but also assists in the repair of periapical tissues. This activity is a result of the presence of a phenolic component stimulating the pulpal fibres in a phenomenon known as hormesis [40,41].

In the present study, calcium hydroxide groups containing either distilled water, glycerin, or both did not have significant antimicrobial effects when compared with groups containing the *O. dubium* essential oil at all time intervals in either the *C. albicans* or *E. faecalis* inoculums (Figure 1). As discussed above, calcium hydroxide is known for its limited activity against *E. faecalis* and *C. albicans* [42]. In this study, the antimicrobial activity of calcium hydroxide increased significantly (*p* < 0.001) with the addition of *O. dubium* essential oil compared with all other study groups at both the 24 h and 48 h measurements (incubations; Figure 1). The data also indicate that the *O. dubium* composition contained 75.8% carvacrol upon GC-MS analysis (Table 2). This might explain the possible increased antimicrobial effect of the calcium hydroxide–*O. dubium* essential oil study group. In a similar study, Nostrat et al. demonstrated the antimicrobial effect of 0.6% carvacrol on *E. faecalis* as an irrigation solution, illustrating its possible effect of disinfecting root canals [25]. The data also revealed that there were no significant differences in the inhibition zone measurements between the groups containing *O. dubium* and *O. dubium*-only, which could be explained by the enormous antibacterial effect of *O. dubium,* given that carvacrol is its major constituent.

The 48 h disk diffusion of *E. faecalis* was reported to be significantly higher, regardless of the different groups and time intervals (Figure 1B). Similarly, the 48 h success rate of a carvacrol-only test group was reported to be 87% in a study by Adel et al. [43]. In another study by Adel et al., carvacrol was found to be effective on its own, and no significant difference was found between carvacrol and calcium hydroxide in bacterial elimination; accordingly, carvacrol was recommended as an alternative intracanal medicament [44]. In the present study, as seen in all groups (Figure 1), the use of carvacrol containing *O. dubium* significantly increased the effectiveness of calcium hydroxide on *E. faecalis*.

Previous studies have demonstrated that carvacrol can impede the growth of different morphological forms of *C. albicans* [45,46,47]. This outcome also correlates with the findings of our study, as groups with *O. dubium* exerted a good range of antimicrobial activity on *C. albicans,* with a larger zone of inhibition when compared with groups without *O. dubium*. The inhibition measurements taken after 72 h of incubation revealed that the addition of *O. dubium* further increased the effect of calcium hydroxide against *C. albicans*. The findings reported by Cacho et al. coincide with the results of our study, as they reported that carvacrol exhibited outstanding potential as a natural compound against *C. albicans* infections [48]. Returning to the present study, the data also revealed that the antimicrobial activity of the group containing calcium hydroxide, glycerin, distilled water, and *O. dubium* was significantly lower than the groups with (1) calcium hydroxide, glycerin, and *O. dubium* and (2) calcium hydroxide, distilled water, and *O. dubium*. This might be a result of the dilution of *O. dubium* in the combination, as the amount applied to each disk belonging to the different groups was equal.

According to the results of a study conducted by Moro et al., carvone was reported to be a significant antifungal agent against *C. albicans* and *Candida*-derived bacteria, with or without Tween 80, which they used for solvent control [49]. In our study, increased antifungal activity was detected as a result of mixing *M. spicata* essential oil, which contained 71.3% carvone and calcium hydroxide. In addition, McGeady et al. reported that carvone exerted an antifungal effect on *C. albicans*, inhibiting the transformation of *C. albicans* into its pathological form [50]. Piras et al. reported that the major component of *M. spicata* was 62.9% carvone, which was found to be 71.3% carvone in our study, depending on the geographical region in which it was collected [51]. Some reports have indicated *M. spicata’s* antimicrobial potential on various bacteria species, excluding *E. faecalis* [52,53], which correlates with the results of this study.

Incubation of a combination of *M. spicata* and calcium hydroxide for 24 h did not show any significant difference in the antifungal effect on *C. albicans* when compared with the use of *M. spicata* alone. However, both groups demonstrated a significantly larger inhibition zone when compared with all other study groups. The study groups containing essential oil, calcium hydroxide, distilled water, and glycerin demonstrated a lower antifungal effect, which could be attributed to the dilution of the concentration of the effective antimicrobial agents. The 72 h incubation data indicate that *M. spicata* and calcium hydroxide exhibited synergistic antifungal activity against *C. albicans.* This could be attributed to the chemicals found in the essential oil composition, which might have induced a faster release of hydroxyl ions in this particular environment.

## 4. Materials and Methods

### 4.1. Plant Material

The plant materials of wildcrafted *Origanum dubium* and cultivated *Mentha spicata* were collected from Lefke, Northern Cyprus, on 10 October 2020 and 21 October 2020, respectively. Aerial parts of the former and the leaves of the latter were dried in shade, resulting in 120 g and 40 g of dried plant material, respectively. The study materials were identified by one of us (K.H.C.B.), and voucher specimens were deposited at the Herbarium of Near East University under voucher numbers NEUN6896 *O. dubium* and NEUN10263 *M. spicata*, respectively.

### 4.2. Isolation of Essential Oil

Dried aerial parts of *O. dubium* (120 g) and dried leaves of *M. spicata* (40 g) were separately hydro distilled with 1 L distilled water for 3 h using a Clevenger-type apparatus. The resulting essential oils were stored at 4 °C until analysis. The oil yields were calculated as *v/w* on a dry weight basis. The essential oil yield of *M. spicata* was 3%, while that of *O. dubium* was 5.5%. 

### 4.3. Gas Chromatography (GC) and Gas Chromatography-Mass Spectrometry (GC-MS) Analysis

#### 4.3.1. GC-MS Analysis

The obtained essential oil compositions were analysed simultaneously by GC-MS analysis. The GC-MS analysis was carried out using an Agilent 5977B GC-MSD system. An Innowax FSC column (60 m × 0.25 mm, 0.25 mm film thickness) was used with helium as the carrier gas (0.8 mL/min). The GC oven temperature was kept at 60 °C for 10 min, programmed to 220 °C at a rate of 4 °C/min, kept constant at 220 °C for 10 min, and then programmed to 240 °C at a rate of 1 °C/min. The split ratio was adjusted to 40:1. The injector temperature was set at 250 °C. Mass spectra were recorded at 70 eV. The mass range was from *m/z* 35 to 450.

#### 4.3.2. GC Analysis

**Figure 4:**
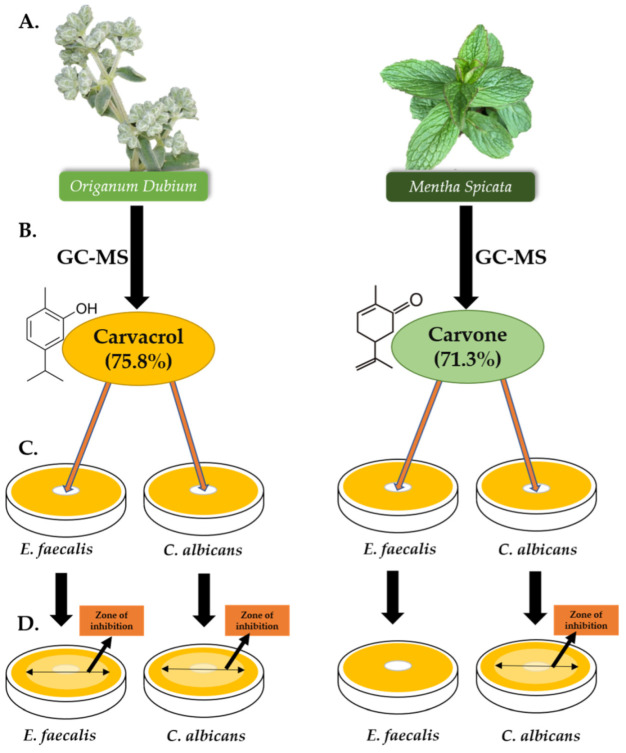
Graphical illustration of the study, with detailed compositions of the most abundant component of each essential oil. (**A**) *O. dubium* and *M. spicata* were collected, and their essential oils were extracted. (**B**) GC-MS analysis of both essential oils were participated. Carvacrol (75.8%) was found to be the most abundant component of *O. dubium* essential oil, whereas carvone (71.3%) was found to be the most abundant component of *M. spicata*. (**C**) Disk diffusion assay was carried out on 2 different microorganisms (*E. faecalis* and *C. albicans*). (**D**) The inhibition zones were measured for *E. faecalis* (24, 48, and 72 h) and for *C. albicans* (48 and 72 h).

The GC analysis was carried out using an Agilent 7890B GC system. The FID detector temperature was 300 °C. To obtain the same elution order used with GC-MS, simultaneous auto-injection was performed on a duplicate of the same column by applying the same operational conditions. The relative percentage amounts of the separated compounds were calculated from the FID chromatograms (Figure 4).

#### 4.3.3. Identification of Compounds

Identification of the essential oil components was carried out by comparison of their relative retention times with those of authentic samples or by comparison of their linear retention index (LRI) with a series of *n*-alkanes. Computer matching against commercial sources (Wiley GC/MS Library, NIST Chemistry WebBook) [54,55] and the in-house “Başer Library of Essential Oil Constituents”, comprising genuine compounds and components of known oils, as well as MS literature data, were used for the identification [56,57].

### 4.4. Preparation of Bacterial and Fungal Cultures

The standard reference of *E. faecalis* used in this study was obtained from the American Type Culture Collection (ATCC 29212) in the microbiology laboratory at Near East University, and the standard reference of *C. albicans* was acquired from the American Type Culture Collection (ATCC 10231) in the same microbiology laboratory (Figure 4). 

### 4.5. Preparation of Experimental Groups

In the present study, seven different study groups were formed. The groups’ ingredients were determined according to the routine protocols applied in the clinic while disinfecting root canals. Calcium hydroxide and distilled water (or saline), together with or separately from glycerin, were added to the powder chemical to prepare the aqueous mixture for washing the canals. In this study, the routine mixtures used in the clinic were also combined with *M. spicata* and *O. dubium* essential oils. A total of seven different experimental groups were formed to investigate the antimicrobial effect of each essential oil together or separately with the routinely applied protocol. For this purpose, equal proportions of different ingredients were combined, as shown in Table 3 for *O. dubium*, and Table 4 for *M. spicata*.

### 4.6. Disk Diffusion Assay

For the disk diffusion test, *E. faecalis* at a concentration of 10^8^ CFU/mL in 100 uL suspension and *C. albicans* at a concentration of 10^6^ CFU/mL in 100 uL suspension were seeded on Mueller Hinton agar plates (Biomerieux, Lyon, France) [58,59].

Seven different study groups containing calcium hydroxide and Cyprus mountain thyme oil (*O. dubium* EO) or garden mint oil (*M. spicata* EO) were tested for antimicrobial activity. For this purpose, the different groups formed were added to the sterile disks to be tested. Distilled water was chosen as a negative control for both microorganisms. Vancomycin was chosen as a positive control for *E. faecalis* and nystatin for *C. albicans*. 

Agar plates for *E. faecalis* were incubated at 37 °C for 24, 48, and 72 h. *C. albicans* was incubated at 37 °C for 48 and 72 h. The inhibition zones formed around the disks on the agar plates indicated the degree of antimicrobial activity (Figure 4). Inhibition zone measurements (mm) were taken at 24, 48, and 72 h for *E. faecalis* and 48 and 72 h for *C. albicans*. Each test was repeated in triplicate to obtain reliable data. 

### 4.7. Statistical Analysis

The inhibition zone diameter data were measured with mean standard errors. The statistical significance of the differences was examined using one-way ANOVA. Tukey’s test was performed for multiple intragroup comparisons, and a *t*-test was performed for intergroup comparisons using GraphPad Prism 5.0 and SPSS for Windows. All *p* values of less than 0.05* were considered statistically significant.

## 5. Conclusions

According to the results of the study, the major compound found in *M. spicata* essential oil was carvone at 71.3%, whereas the major compound found in *O. dubium* essential oil was carvacrol at 75.8%. *M. spicata* essential oil was found to be ineffective against *E. faecalis,* whereas it demonstrated a superior effect on *C. albicans* at 72 h when combined with calcium hydroxide. The effect of *O. dubium* essential oil activity could be based on the enormous amount of carvacrol it contains, which induces bacterial membrane damage by decreasing the intracellular ATP level and increasing the proton permeability of the membrane, which results in the leakage of K+ out of the bacterial cell as an indicator of membrane damage [60]. The effect of *M. spicata* could be attributed to the rich content of carvone, which has the capacity to penetrate bacterial cells, disturbing cell membrane permeability and hence its integrity [61]. With respect to *O. dubium* oil, it was found to have very effective antimicrobial activity against both *E. faecalis* and *C. albicans.* Altogether, the data reveal that the usage of these essential oils with calcium hydroxide as intracanal medicaments exerted good antimicrobial effects and that they could be promising candidates for use in routine clinical endodontic treatments in the future.

## Figures and Tables

**Table 1:** The essential oil composition of *M. spicata*.

	LRI	Compound Name	Relative Percentage Amount (%)
1	1015	α-Pinene	1.0
2	1068	Camphene	0.1
3	1115	β-Pinene	1.2
4	1128	Sabinene	0.8
5	1168	Myrcene	0.8
6	1207	Limonene	12.5
7	1217	1,8-Cineole	3.6
8	1241	β-Terpinene	0.2
9	1256	γ-Terpinene	0.1
10	1259	(*Ε*)-β-Ocimene	0.1
11	1393	3-Octanol	0.2
12	1539	β-Bourbonene	0.4
13	1599	Camphor	0.1
14	1608	β-Elemene	0.4
15	1618	Terpinen-4-ol	0.2
16	1623	β-Caryophyllene	0.5
17	1635	*trans*-Dihydrocarvone	0.3
18	1663	*cis*-*iso*-Dihydrocarvone	0.1
19	1676	Pulegone	1.1
20	1687	Dihydrocarvyl acetate	0.6
21	1699	Bicyclosesquiphellandrene	0.4
22	1721	Borneol	0.2
23	1739	Germacrene D	0.6
24	1768	Carvone	71.3
25	1789	*p*-Vinyl anisol	0.9
26	1819	α-Cadinene	0.1
27	1828	*cis*-Carvone oxide	tr
28	1851	*trans*-Carveol	0.3
29	1863	*cis*-Calamenene	0.2
30	1867	*trans*-Calamenene	0.1
31	1882	*cis*-Carveol	1.4
32	2090	Cubenol	0.2
33	2260	Torreyol	0.1
Total			100.0

LRI: Linear retention indices calculated against *n*-alkanes. %: calculated from FID data. tr: Trace (<0.1%).

**Table 2:** The essential oil composition of *O. dubium*.

	LRI	Compound Name	Relative Percentage Amounts (%)
1	1015	α-Pinene	3.1
2	1019	α-Thujene	0.9
3	1067	Camphene	0.2
4	1114	β-Pinene	0.2
5	1155	δ-3-Carene	0.9
6	1167	Myrcene	1.4
7	1172	β-Terpinene	0.2
8	1187	α-Terpinene	1.2
9	1206	Limonene	0.3
10	1216	1,8-Cineole	0.2
11	1217	β-phellandrene	0.3
12	1255	γ-Terpinene	4.1
13	1283	*p*-Cymene	6.9
14	1294	Terpinolene	0.3
15	1451	1-Octen-3-ol	0.1
16	1472	*trans*-Sabinene hydrate	0.4
17	1549	Linalool	0.2
18	1558	*cis*-Sabinene hydrate	0.2
19	1618	Terpinen-4-ol	0.8
20	1622	β-Caryophyllene	0.4
21	1710	α-Terpineol	0.6
22	1715	γ-Terpineol	0.2
23	1720	Borneol	0.3
24	2027	*neo*-*iso*-Dihydro carveol	0.2
25	2151	Spathulenol	0.1
26	2199	Thymol	0.5
27	2231	Carvacrol	75.8
Total			100.0

LRI: Linear retention indices calculated against *n*-alkanes. %: calculated from FID data. tr: Trace (<0.1%).

**Table 3:** Composition of *O. dubium*, calcium hydroxide, distilled water, and glycerin between different study groups.

	*O. dubium* E.O.	Calcium Hydroxide	Distilled Water	Glycerin	
*Origanum dubium* EO and Calcium Hydroxide	100 μL	0.001 g	-	-	The proportion of group ingredients
*Origanum dubium* EO and Distilled water and Calcium Hydroxide	100 μL	0.002 g	100 μL	-	
*Origanum dubium* EO and Glycerin and Calcium Hydroxide	100 μL	0.002 g		100 μL	
*Origanum dubium* EO and Distilled water and Glycerin and Calcium Hydroxide	100 μL	0.003 g	100 μL	100 μL	
Distilled water and Calcium Hydroxide	100 μL	0.001 g	-	-	
Glycerin and Calcium Hydroxide	-	0.001 g	100 μL	-	
Distilled water and Glycerin and Calcium Hydroxide	-	0.001 g		100 μL	

**Table 4:** Composition of *M. spicata*, calcium hydroxide, distilled water, and glycerin between different study groups.

	*Mentha spicata* EO	Calcium Hydroxide	Distilled Water	Glycerin	
*Mentha spicata* EO and Calcium Hydroxide	100 μL	0.001 g	-	-	The proportion of group ingredients
*Mentha spicata* EO and Distilled water and Calcium Hydroxide	100 μL	0.002 g	100 μL	-	
*Mentha spicata* EO and Glycerin and Calcium Hydroxide	100 μL	0.002 g		100 μL	
*Mentha spicata* EO and Distilled water and Glycerin and Calcium Hydroxide	100 μL	0.003 g	100 μL	100 μL	
Distilled water and Calcium Hydroxide	100 μL	0.001 g	-	-	
Glycerin and Calcium Hydroxide	-	0.001 g	100 μL	-	
Distilled water and Glycerin and Calcium Hydroxide	-	0.001 g		100 μL	

## Data Availability

Not applicable.
